# Manipulation cases in free will and moral responsibility, part 2: Manipulator‐focused responses

**DOI:** 10.1111/phc3.70008

**Published:** 2024-11-27

**Authors:** Gabriel De Marco, Taylor W. Cyr

**Affiliations:** ^1^ University of Oxford Faculty of Philosophy Uehiro Oxford Institute Oxford UK; ^2^ Department of Classics and Philosophy Samford University Birmingham Alabama USA

## Abstract

In this paper—Part 2 of 3—we discuss one of the two main types of soft‐line responses to manipulation cases, which we refer to as manipulator‐focused views. Manipulator‐focused views hold, roughly, that the reason that Victim lacks responsibility (or lacks full responsibility) is because of the way the action is related to the Manipulator. First, we introduce these views generally, and then we survey some detailed versions of such views. We then introduce cases of natural forces, often taken to be a problem for such approaches in general, followed by a discussion of various sorts of cases—accidental results, lucky manipulators, and parallel cases—that present challenges for some of the detailed versions of such views. We conclude with some thoughts about the prospects for manipulator‐focused views going forward.

## 1 INTRODUCTION

In the first paper in this series (which will be helpful background for this paper), we introduced various cases of manipulation, the manipulation argument against compatibilism, and a handful of important issues in debates about manipulation in the literature on free will and moral responsibility, including the hard‐line response (according to which Victim is free and responsible). In this paper and its sequel, we survey proposals that fit into the two main types of *soft‐line* responses (which accept that Victim lacks responsibility for their action, and offer an account of the difference between Victim and a standard agent). In this paper, we consider *manipulator‐focused* views, and the sequel covers *bypassing* views.

Recall two cases of manipulation introduced in Part 1 (condensed here):

*Sweet Sally*: When [Sweet] Sally crawled into bed last night, she was one of the kindest, gentlest people on Earth…But Sally awakes with a desire to stalk and kill a neighbor, George….What happened is that, while Sally slept, a team of psychologists…implanted [new] values in Sally after erasing her competing values…Seeing nothing that she regards as a good reason to refrain from stalking and killing George, provided that she can get away with it, Sally devises a plan for killing him; and she executes it—and him—that afternoon…

(Mele, 2019: 20–1)

*Ernie*: Diana, a goddess with special powers and knowledge, wants event E to occur 30 years after time t. Diana…creates a zygote, Z, in Mary at time t which will develop into Ernie. Diana does this knowing that, given the state of the world and the laws, Ernie will, 30 years in the future, perform action A which will bring about event E.

(Mele, 2006: 188–9)

*Sweet Sally* is a case of mid‐life manipulation, and *Ernie* is a case of original design. Both cases feature a Victim (Sally, Ernie) as well as a Manipulator (psychologists, Diana).

Manipulator‐focused views hold, roughly, that the reason that Victim lacks responsibility (or lacks full responsibility) is because of the way the action is related to the Manipulator. Some authors suggest that at the very least, the presence of the manipulator is affecting our intuitions.^1^ Others have suggested that the relevant difference between Victim and a standard agent might be that Victim is Manipulator's puppet, extension, or tool (Herdova, 2021; Ismael, 2016, p. 104; Kearns, 2012; Lycan, 1987, p. 117) or that Manipulator, rather than Victim, is the casual source of the action (Deery & Nahmias, 2017). Relatedly, one might think that being subject to intended or intentional manipulation (Barnes, 2015; Ragland, 2011, p. 68; R. R. Waller, 2014) or the control of Manipulator (Seybold, 2022; Usher, 2020), or being “under the sway” of the Manipulator (Lee, 2024, p. 20) can undermine, or at least mitigate, responsibility. One might instead argue that Victim has been “rigged to interact with the circumstances” so that they end up acting as they do (Schlosser, 2015, p. 80) or that Manipulator is tracking what goes on inside of Victim, and thus fewer lives are available to Victim (Yaffe, 2003). Alternatively, one might argue in favor of a manipulator‐focused view more generally, without offering a specific account (Liu, 2022).^2^

Our approach in this article will be as follows. First, we briefly introduce specific, more developed, versions of manipulator‐focused proposals. We then return to the bigger picture, and consider potential issues for this type of view.

Before moving to these views, however, we make a couple of general points. First, some of these views are stated in terms of the freedom required for moral responsibility (Yaffe, Barnes), one of these is simply about responsibility (Waller), and some of these discuss both (Herdova, Deery and Nahmias). For ease of presentation, we discuss them all in terms of (moral) responsibility.^3^ Second, as we initially described soft‐line views, they accept the claim that Victim lacks responsibility (and freedom) for the relevant action. Yet this is not quite right for some of the views discussed below. We make it clear when we introduce each view, but some only offer an account of Victim's *mitigated* responsibility, some suggest it might mitigate, and potentially *eliminate*, responsibility, and a couple alternate between claiming that Victim lacks responsibility, and that they lack *full* responsibility.

## 2 MORE DETAILED PROPOSALS

### 2.1 Yaffe

Though Yaffe (2003) mainly considers cases which are somewhat different than the ones we have discussed so far, he offers a view intended to explain why manipulation undermines responsibility. In typical cases, Manipulators track the production of a pattern of responding to reasons in Victim, and are ready to (successfully) intervene if this production goes off‐course. For standard, unmanipulated, agents however, there is no‐one tracking their pattern of response to reasons, and if they go “off‐course”, they simply end up with a different pattern of responding to reasons. Because of this, Yaffe suggests, Manipulators “limit our options in ways that neutral causal forces do not” (2003: 344), and for Victims, “fewer lives are available” (2003: 345). This implies that their responsibility is, at least, mitigated.^4^

### 2.2 Barnes

Building on Yaffe's work, Barnes (2015) develops a manipulator‐focused view according to which manipulators deprive their victims of creativity and thus of (full) responsibility.^5^ For an agent to act creatively (and thus be responsible), “there [must] be at least one nearby possible world (which may be the actual world) in which he exhibits communal creativity by acting in a way which his community has not communicated to him” (2015: 568).^6^ Barnes continues:

I offer the following clarification of what it means for a community to communicate an action X to B: (1) A (a member(s) of B's community) grasps the idea of X and (2) intends for B to grasp the idea of X and (3) performs actions that serve to transmit information constituting the idea of X from A to B.

(2015: 568)

The view is developed with an eye toward multiple versions of the Ernie case. But, assuming that Diana meets (3), the view extends to mid‐life manipulation cases too.^7^ In both types of case, Manipulators are part of Victim's community because they are “engaged in the direct shaping of [Victims'] cognitive and behavioral development” (2015: 572), and they communicate the pertinent action to their victims. Further, they do this in possible worlds that count as nearby as well.

### 2.3 Waller

Waller proposes a manipulator‐focused view with some similarities to Barnes's, though hers focuses on the *effective intentions* of manipulators and the mitigating effects they have on what praise/blame their victims deserve. As Waller suggests, “effective intention” could be interpreted in different ways. On a weak interpretation, the claim that an agent had an effective intention to A entails the claim that A occurs (and, presumably, that the intention was among the causes of A). On a stronger interpretation, it also implies that the agent intentionally A‐ed (2014: 212‐3). She does not commit to either, but we interpret her view as using the weaker interpretation, and return to the stronger reading toward the end of the paper. The view is as follows:

T*: S is less deserving of blame or praise for A‐ing than she would be otherwise if

(1) another agent G effectively intends that S A‐s,
(2) G brings it about that S A‐s via intervention, and
(3) S did not intentionally bring it about that G intends that S A‐s (R. R. Waller, 2014, p. 216).

Like Barnes, Waller focuses on the Ernie case, but her conditions (1)‐(3) are satisfied by Victims and Manipulators in mid‐life manipulation cases as well, yielding the conclusion that these Victims lack full responsibility.^8^

### 2.4 Herdova

Herdova (2021), mainly focusing on cases of original design like that of Ernie, proposes that one difference between Ernie and ordinary determined agents is that the former is the *tool* of Diana, whereas ordinary determined agents are not the tools of other agents. This difference in toolhood is plausibly a responsibility‐relevant one; being someone's tool might mitigate one's responsibility, and potentially even eliminate it. Herdova suggests a non‐exhaustive list of criteria for A's being the tool of B—e.g., B intentionally, covertly and non‐consensually ensures that A acts in certain ways, or B has a vested interest in A acting in those ways—and says that, “when enough of these conditions are present, they can collectively contribute to the extent to which someone is the tool of another” (2021: 259). Many, if not all, of the criteria on her list are satisfied by Manipulator and Victim in typical cases of original design and mid‐life manipulation. Thus, these Victims are Manipulators' tools and lack (full) responsibility.

### 2.5 Deery and Nahmias

Another route to a manipulator‐focused view implements resources from *interventionist theories of causation* (Woodward, 2009, 2023) in order to assess manipulation cases. We focus on such a view as developed by Deery and Nahmias (2017), though Usher (2020) offers one as well.^9^

Deery and Nahmias (DN) develop an account of causal sourcehood, and suggest that an agent must be the causal source of an action—or, the causal source must lie within the agent—in order to be, at the very least, fully responsible with respect to it.^10^ And in order to be the causal source of an action, one must bear the strongest causal invariance relation to the action (2017: 1263), though there may be ties for strongest. The strength of the causal invariance relation is measured by two features of causal relations described in at least some interventionist theories of causation. We will follow Tierney and Glick (2020) in referring to these as *stability* and *reliability*. Here, we simply offer a very brief, and rough, sketch of these relations.

Interventionist views make use of a causal model to evaluate causal claims relating three variables—the outcome to be assessed (O), the potential cause (C), and the background conditions (B)—by seeing what would happen to the outcome variable when changes are made to the other variables. Consider *reliability*, which we can evaluate by making a change to the value of a potential cause (C)—say Ernie's decision to A—without making any changes to any of the usual causes of the decision, or to any of the background conditions. We can then see if this results in a change in the value of the outcome (O)—say, Ernie's A‐ing.

Or compare, for instance, an expert sharpshooter with a novice who both hit a bulls‐eye in the dead‐center—one of them through skill, the other through luck. A change in where the expert sharpshooter decides to hit (C) will be a much better predictor of where the bullet hits (O)—holding all else fixed—when compared to a change in the novice's decision; the sharpshooter's decision is much more *reliably* related to where the bullet hits than is the novice's.

A second feature of causal relations is *stability*. To borrow a case from Usher, compare shooting a person in the leg to shooting a person in the heart, when there is no medical help readily available in either case (2020: 308–9). Both shootings, we can suppose, cause the victims' deaths (O). Yet shooting a person in the leg is more sensitive to changes in background conditions (B) than shooting them in the heart, and this is because there are many more ways of changing the background conditions—e.g., the availability of medical help—which can change whether the victim dies. The causal relationship between shooting the victim in the heart and the victim's death is more *stable* than that between shooting another victim in the leg and their death.

Now recall that, on DN's view, the casual source of an action is that which bears the strongest causal invariance relation to it. On DN's view, a potential cause, C1, has a stronger causal invariance relation to an outcome, O, than some other cause, C2, if and only if C1 is more stably and more reliably related to O than is C2 (2017: 1262–3). Agents performing intentional actions are often the causal source of their actions; what they intend to do is often a good predictor of what they will do, and unlike non‐agents, they are able to track features of their environments that are relevant to achieving their goals; that is, they can adapt their behavior to the background conditions that are relevant to their achieving their goal.

Things get complicated, however, once more than one agent is involved, as in standard cases of manipulation. With a focus on the case of Ernie, DN suggest that Diana is the causal source of Ernie's A‐ing since Diana's decision that Ernie A (C1) bears a stronger causal invariance relation to Ernie's A‐ing (O) than does Ernie's decision to A (C2). Thus, Ernie lacks (full) responsibility for his A‐ing (2017: 1264–5). Such a result plausibly extends to cases of mid‐life manipulation like that of *Sweet* *Sally*.^11^

We turn to general objections to manipulator‐focused views shortly, but before moving on, we mention a criticism of interventionist views in particular, offered by Tierney and Glick (2020), with a special focus on DN's view.^12^ Recall that on this view, an agent—including Victim—lacks (full) responsibility when the casual source lies outside of them insofar as Manipulator's decision (or intention) is *both* more stably *and* more reliably related to the outcome than Victim's.

Tierney and Glick point out that this view will have difficulty handling cases in which any potential cause that is most stably related to the outcome is different from any that is most reliably related to it. Consider the following, paraphrased from Tierney and Glick (2020: 959):

*Mob Hit*: A mafia boss decides to hire an assassin to kill an enemy and does so. The assassin decides to kill his target by poisoning his dinner Thursday evening and does so.

The assassin's decision seems to be more *reliably* related to the murder than the boss's decision, insofar as we can make more changes to the assassin's decision that predict the value of the outcome (the murder) than we can to the boss's. Though the boss can plausibly only decide whether to hire an assassin, or which one, the assassin decides the means, day, time, etc. of the murder. But, the Boss's decision is more *stably* related to the murder. For instance, were the assassin to fail due to changes in background conditions, the boss could still hire another assassin to finish the job. Since neither decision is *both* more stably and more reliably related to the murder than the other, neither has the stronger causal invariance relation.

On DN's view, it seems that neither decision is the casual source, leading us to the awkward conclusion that neither is responsible for the murder (on the assumption that causal sourcehood is necessary for responsibility *simpliciter*). Tierney and Glick then go on to consider different ways of revising or expanding the view, posing issues for each of these.

Here, we briefly suggest that Tierney and Glick's point might be expanded to some standard cases of manipulation. It would not make a significant difference to the case, we suspect, if we stipulate that Manipulators in *Sweet Sally* are relevantly similar to Tierney and Glick's mafia boss. If so, and *Mob Hit* poses issues for a view like DN's, then this slight variation of *Sweet Sally* will as well, and the view faces an issue with accounting for Victim's lack of (full) responsibility in a relatively standard case of mid‐life manipulation.

## 3 GENERAL CHALLENGES FOR MANIPULATOR‐FOCUSED VIEWS

Which challenges particular views face will depend on the details. We begin, however, with one challenge that all such views seem to face.

### 3.1 Natural forces

The most commonly raised challenge to such views can be seen with *natural force* cases. Consider the following:

*Natalie*: Before she went to bed last night, Natalie was, like Sally, one of the kindest, gentlest people on Earth. However, due to a strange electro‐magnetic storm over her house while she slept, Natalie underwent a change just like the one Sally underwent. Upon waking, Natalie devises a plan for killing her neighbor; and she executes it—and him—that afternoon.^13^

This case is very similar to (indeed, based on) a case of mid‐life manipulation, and many who find Victim not (fully) responsible in that sort of case will judge Natalie to lack (full) responsibility as well. Yet, since the change to Natalie results from natural forces and not from another agent, manipulator‐focused views cannot account for her lack of (full) responsibility.

Several authors we have discussed above do not explicitly address this worry.^14^ One exception is DN, who accept that agents in natural force cases do not have mitigated responsibility, writing that if “there is no intentional manipulator, but just a factor that is stipulated to causally influence an agent's later decision, then our metric of causal sourcehood explains why the hardline response is appropriate in those cases” (2017: 1273).^15^ Yaffe (2003: 345‐6) is another exception. He divides natural force cases into one of two categories, and suggests that neither type of case poses a challenge. First, if the natural force is functionally similar to responsibility‐undermining manipulation (as in his example of the “robotic tutor”), then the natural force is responsibility‐undermining as well, and for the same reasons. Second, if the natural force is not functionally similar to responsibility‐undermining manipulation—if nothing is tracking the production of a pattern of response to reasons in Victim—then there is no problem with accepting that responsibility is not undermined in the case.^16^

### 3.2 Accidental results

These views tend to be developed with an eye toward cases in which Manipulator is successful, and focus on Victims' actions that were intended by Manipulator. Yet a challenge might arise once we modify these features of the case(s). Consider some variations of mid‐life cases in which Manipulators cannot foresee—and do not intend—certain results of the manipulation. For example, consider a variant of *Sweet Sally* in which Sally kills an additional person (whom Manipulators did not know about), or in which, due to a mistake by Manipulators, Sally kills a different person than Manipulators' intended target.^17^ One might think that just like Sally lacks (full) responsibility in the original case of *Sweet Sally*, so does Sally in these variations.

Yet, insofar as the Manipulators in these variations do not intend the accidental results, some manipulator‐focused views cannot account for Victim's lack of (full) responsibility. Manipulators do not communicate these actions to Victim in Barnes's sense, for example, nor is Victim's behavior effectively intended by Manipulators in Waller's sense. And whereas interventionist views like DN's do not take the Manipulators' communication or intentions to be necessary to accounting for Victim's lack of (full) responsibility, the results being different than (indeed, contrary to) Manipulators' intentions would seem to count against their intentions being the causal source of these actions, as the causal relations between Manipulators' intentions and Victim's actions are less stable and/or reliable than the causal relation than in the original *Sweet Sally*.

Yaffe's view may initially have an advantage here. After all, even in these variations, the Manipulators were tracking the production of a certain pattern of recognition of reason in the respective Sallys, and their murders are a result of this. Yet consider a variation in which Manipulator's mistake is even bigger: instead of their surgery resulting in Sally murdering her neighbor, she ends up joining a traveling theater troupe (which she had not previously considered and is radically different from anything she had ever done). Yaffe's view, in this case, would not apply. This last case brings us to a related challenge, arising from lucky Manipulators.

### 3.3 Lucky manipulators

Absent perfection, everyone—even a highly skilled Manipulator—can make a mistake. And absent perfection, everyone—even a Manipulator with little‐to‐no skill—can be lucky to succeed. Unskilled Manipulators, however, cannot *ensure* that Victims will act in a particular way. Thus, consider:^18^

*Manipulating Fledgling*: 20 years before he manipulated Sally, our neurosurgeon was just a fledgling who decided to begin learning how to perform this sort of surgery. He begins by trying to turn an equally sweet person, Nelida, into someone as evil as post‐manipulation Sally. It works: Nelida wakes up, reflects on her new values, and decides to kill Juan, her neighbor. When he tries to do this again on other subjects, he fails miserably. Most of them die on the operating table, and the rest end up with strange results: an obsession with petting penguins, feeling extreme pangs of pain every time she sees corduroy, or thinking of oneself as a kite floating over the Sahara. His success with Nelida was not a pure stroke of genius, but rather a pure stroke of luck.

If, as with Sally, one thinks Nelida lacks (full) responsibility, then some manipulator‐focused views will face difficulty in accounting for this thought, despite the presence of Manipulating Fledgling (MF).

Lucky MF has a goal, but he fails to track the production of a pattern of responding to reasons, given that he has little‐to‐no skill. Consequently, Yaffe's explanation of why Victims lack responsibility does not apply here.^19^ And this lucky MF would not seem to be the causal source of Nelida's action—in DN's sense—since Nelida's decision to kill her neighbor is more stably and reliably related to the murder than is MF's decision (or intention).^20^

Waller's view, on the other hand, could get us the claim that Nelida is less deserving of blame, since MF still effectively intended that Nelida killed her neighbor, and brought this about via an intervention. Things are less clear with Barnes's and Herdova's views. Given his inability to *ensure* that Nelida act in this way, several of Herdova's criteria for toolhood are not met. And depending on which worlds are nearby—on Barnes's metric—there may be nearby worlds in which Nelida acts creatively.

### 3.4 Parallel cases

Finally, recall a type of case we mentioned in Part 1: *parallel cases*—cases where manipulated agents meet conditions on responsibility for an action posited by at least some incompatibilist views.^21^ Thus, suppose that sweet Sally lives in a world that is not deterministic, and that at the time at which she decides to kill her neighbor George (or shortly before), it is open, given the past and laws, that she would instead decide to kill his son. Had no manipulation occurred, neither option would have occurred to Sally.

We find it plausible that Sally is not (fully) responsible for killing George. However, the indeterminacy would seem to prevent the manipulators from being more of a causal source than Sally. The output of Sally's CAS in this case—her decision to kill George—would seem to be causally related to her killing of George in a more stable and reliable way. And supposing that a world with the same past and laws, in which Sally decides to kill George's son instead, is nearby on Barnes's metric, she would seem to be responsible.

As with *Manipulating Fledgling*, this case is also not clearly a problem for some other manipulator‐focused views. Waller's view, for example, might still get us the result that Sally lacks full responsibility for killing George, insofar as the Manipulator's intention was effective. However, consider a slight variation of the case. Suppose that everything is the same, except that Sally decides to kill George's son instead. In this case, Manipulators' intention that Sally kill George is not effective.

Of the views surveyed above, we think, Yaffe's is the only one that may be able to account for both parallel Sallys' lack of (full) responsibility. In both versions, Manipulators successfully induce the pattern of response to reasons that they would seem to be tracking, since they are trying to make her like Chuck; and this holds regardless of who she decides to kill.

## 4 CONCLUDING THOUGHTS

Detailed versions of manipulator‐focused views are, with the exception of Yaffe's view, relatively new additions to the debate, and different views face different challenges. We can, however, conclude with a few general comments.

In standard cases of manipulation—like *Sweet Sally* or *Ernie—*Victim's actions are related to Manipulator in a variety of ways. For example, Manipulator intends that Victim perform some action, that intention is effective, and Manipulator achieves this via intentional and highly skilled action, with knowledge of a large amount of features relevant to the adequate execution of their intention. These features help make it such that Manipulator has, and exercises, a great amount of control over Victim's behavior. And manipulator‐focused views are able to account for Victim's lack of (full) responsibility by capturing at least some of these features in some way or other; for example, by incorporating the effective intention into the view, by developing an account of causal sourcehood, or by requiring that the manipulator *track* the production of a certain pattern in Victim.

Actions performed by agents in standard natural force cases—like Natalie's action—lack *all* of these features, since such cases lack a Manipulator altogether. Yet as we have seen, some proponents of manipulator‐focused views are willing to accept that agents like Natalie are (fully) responsible for their actions. Someone willing to take this line, however, still faces issues with other cases, like the different versions of accidental results cases, lucky manipulators, and parallel cases.

Some of these versions of manipulation cases retain the presence of a “manipulator”, yet Victim's relevant action is not related to Manipulator in any of the ways mentioned above—like the accidental results case in which Sally kills a further person Manipulators did not know about, or in which she ends up joining a theater troupe. Other versions pry apart some of these features. For instance, lucky Manipulators effectively intend their Victim's action, despite their lacking great skill, and plausibly not even intentionally achieving their results.^22^

We conclude by focusing on two of these features: Manipulator's *intending* that Victim perform some action, and Manipulator's *skillfully* (and intentionally) achieving their intended result. Various manipulator‐focused views incorporate these (or something highly related) in their conditions, but two exceptions stand out. The weak version of Waller's view requires the former and not the latter, insofar as an intention can be effective for different reasons (e.g., it can deviantly cause the relevant behavior). And Yaffe's view requires the latter, but not clearly the former, insofar as what Manipulator must “track” is the production of a pattern of responding to reasons, but maybe not any particular action by Victim.

These unique features of the accounts confer advantages, but also come with costs. Focusing mainly on the effectiveness of the intention allows Waller's view to account for Victim's lack of (full) responsibility in cases involving lucky manipulators, as well as one version of a parallel case. Yet it still faces issues with other variations.

Further, it seems to imply that we lack (full) responsibility for ordinary actions in social contexts, like when a friend gently persuades us to do something, or if we attend a party on the basis of a sincere invitation. Importantly, adding a feature like this to standard cases of manipulation—say, Ernie's friend suggests that Ernie A with the intention that he does so (which Diana incorporated as part of her plan)—means that the view cannot account for a relevant difference between *this* Ernie and a standard agent in a deterministic universe who does the same thing, and has a past identical to Ernie's, yet whose zygote came about in the normal way.^23^ This result could be avoided by incorporating a skill component, yet this runs the risk of losing some of the advantages, like the ability to account for Victim's lack of (full) responsibility in *Manipulating Fledgling*.

Yaffe's view, on the other hand, faces issues with cases like *Manipulating Fledgling* insofar as MF's lack of skill prevents him from adequately “tracking” the production of a pattern of responses to reasons. Yet it has advantages with respect to other cases, like both parallel versions of Sally, and some versions of accidental results cases. A large reason for this, we think, is that it does not require that Manipulator intend that Victim perform any *particular* action. What matters is the pattern of response to reasons, which stays with Victim even after they perform the action Manipulator intended (in cases where Manipulator has such an intention) and can be expressed in a variety of other actions.

The final article in this series, Part 3, focuses on bypassing views. None of these views rely on features of Manipulator in explaining Victim's lack of responsibility, and all of them share this relevant feature with Yaffe's view.

## CONFLICT OF INTEREST STATEMENT

The authors declare no conflict of interest.

## ENDNOTES

^1^
For early examples, see (Dennett, 1984, p. 8; B. N. Waller, 1988, p. 156), and the discussion of hard‐line responses in Part 1.

^2^
For some other examples, see (Berofsky, 2006a, 2006b). For some examples of a manipulator‐focused view with respect to some sense of freedom, but perhaps not the sense relevant to responsibility, see (Garnett, 2015; Gingerich, 2022).

^3^
See also Section 3 of Part 1.

^4^
Haji and Cuypers (2008, p. 199) argue that this view would get us the wrong result in cases of education, insofar as a teacher may be tracking the production of a particular pattern of responding to reasons in their students. For further discussion, see Haji and Cuypers (2008, pp. 199–201) and Yaffe (2003, pp. 348‐9).

^5^
Barnes sometimes suggests that his condition is necessary for an agent's being *fully* free (e.g., 2015: 567), and sometimes that it is necessary for an agent's being free at all (e.g., 2015: 568).

^6^
For Barnes's elaboration on a world's being nearby, see Barnes (2015, p. 562).

^7^
Though it is hard to see how Diana satisfies (3) in the original version of *Ernie* or how Manipulator communicates anything to Sally by fiddling with her neurons.

^8^
For discussion of the view, see Whittle (2021, pp. 188–90). For a somewhat similar view, see Coates (2019).

^9^
For further discussion of Usher's view, see n. 11 below. A different view making use of a similar framework, though not clearly designed for manipulation cases in particular, is offered by Murray and Lombrozo (2017).

^10^
DN do not specify whether this statement is to be restricted to *direct* responsibility, but that is how we will read them. Also, they sometimes speak of causal sourcehood as necessary for freedom and responsibility simpliciter (2017: 1267), and other times, only as necessary for being *fully* free or responsible (2017: 1260, n. 6).

^11^
We can now briefly say something about Usher's view (2020). Very roughly, his view focuses just on the stability of the causal relation between the agent's intention and their action, while also requiring—for what he calls *teleological control*—that the agent is online and monitoring the situation and is ready and able to intervene if necessary. And, again roughly, if one agent exercises teleological control over another, then the latter lacks (full) responsibility. As Usher notes, his view cannot account for Ernie's lack of (full) responsibility given that Diana is not online at the time of Ernie's action (2020: 320). A couple of points are worth making about this response. First, Tierney and Glick suggest that Usher is wrong to take this line and ought to claim that Diana's presence at least reduces Ernie's responsibility (2020: 958, n. 6). In fact, he does seem to take a soft‐line view with respect to Pereboom's case 2 (2020: 318), which is often interpreted as not relevantly different from the case of *Ernie*. Second, this response to *Ernie* is quite pertinent, insofar as Manipulators in mid‐life cases—like that of Sally—can plausibly be understood as failing to be online at the relevant time as well.

^12^
In a separate paper, Tierney (2023) offers a further criticism of such views. Roughly, the point is that there is something illicit, circular, or at least uninformative about using intuitions about free will to inform our account of causation, which in turn will be used to inform our account of free will (2023: 439).

^13^
For discussion of such cases, see (Arpaly, 2006, pp. 111–12; Barnes, 2015, p. 564; Brink, 2021, p. 100; Caruso, 2021, p. 85; De Marco, 2023; De Marco & Cyr, 2024; Deery & Nahmias, 2017, p. 1272; Fischer, 2011: 270, 2016: 50; Liu, 2022; Mele, 1995, pp. 168–9, 2006: 141, 2019: 27, 31, 58, 94, 155, n. 10; Mickelson, 2019, p. 171; Nelkin, 2011, pp. 57–9; Pereboom, 1995: 25, 2001: 115, 2014: 79, 82; Sartorio, 2016, pp. 160–1).

^14^
Barnes (2015) considers natural force cases too but, like Sartorio (Sartorio, 2016, pp. 160–3), only the initial design variation, and the mid‐life case of *Natalie* remains a problem for Barnes's view.

^15^
DN (2017: 1272) also say that there can be cases in which a natural force (such as ambient noise) could be the causal source of an agent's action and thereby mitigate the agent's responsibility. Yet, standard cases of natural forces—like *Natalie*—are not like this.

^16^
It is also worth noting that Liu (2022) argues that natural force cases are not a problem for manipulator‐focused views but that they give rise to a dilemma for bypassing views (and so ultimately favor manipulator‐focused views). Liu also discusses and criticizes DN's approach (2022: 541–543). See De Marco (2023) for a critical response to Liu.

^17^
For a more detailed discussion of such cases, as well as the issues they pose for particular views, see De Marco and Cyr (2024). For related cases employed against Yaffe's view, in which Manipulators are indifferent (and thus not tracking the production of a particular pattern of reasons), see Barnes (2015, pp. 564‐5) and Haji and Cuypers (2008, p. 198).

^18^
For discussion of lucky manipulators, and their implications for manipulator‐focused views, see (Caruso, 2021, pp. 88–9; De Marco & Cyr, 2024; Deery & Nahmias, 2017, p. 1273; Pereboom & McKenna, 2022, pp. 192–3; Tierney, 2023, p. 448, n. 8; Tierney & Glick, 2020, pp. 958–9, n. 7; Yaffe, 2003, pp. 346–8).

^19^
Though see his discussion of the weakling monarch (2003: 346‐8).

^20^
DN consider this sort of case along with that of natural forces, and offer the same response. After doing so, DN appeal to studies (Murray & Lombrozo, 2017; Phillips & Shaw, 2015) that seem to show that “people are far less likely to have the intuition” (or reduced responsibility) if the manipulator is like a physical event (2023: 462). Although we cannot get into the details here, we think there are (at least) two potential factors that could explain a difference in judgments of responsibility between the relevant cases, and so we do not think the studies are conclusive in DN's favor. See (De Marco & Cyr, 2024, p. 20, n. 38) for discussion.

^21^
See, for example (Clarke, 2012; Cyr, 2016, 2020; De Marco & Cyr, 2024; Haji & Cuypers, 2001; Harrison, 2008, 2010; King, 2013; McKenna, 2016, pp. 87–8; Mele, 2006, p. 52; Pereboom, 2001, pp. 41–54, 2014,: 47–8, 84, n. 7; van Inwagen, 1983, p. 132).

^22^
Another sort of case we do not discuss here are Frankfurt cases (Frankfurt, 1969). In these cases, a powerful would‐be intervener ends up *not* intervening to ensure would‐be Victim acts in a certain way, since no such intervention is necessary. The would‐be intervener here has the relevant intention, and a significant amount of skill related to the robust properties that, say DN ask for in causal sourcehoood. For some discussions, see De Marco and Cyr (2024).

^23^
For further discussion of this point, see De Marco and Cyr (2024, pp. 9–10).
